# Serum endocan level and diastolic functions in the case of lead exposure

**DOI:** 10.3906/sag-1801-146

**Published:** 2019-02-11

**Authors:** Halil KARA, Uğur Nadir KARAKULAK, Meside GÜNDÜZÖZ, Ceylan BAL, Murat ALIŞIK, Murat BÜYÜKŞEKERCİ, Servet Birgin İRİTAŞ, Omer HINÇ YILMAZ, Lütfiye TUTKUN

**Affiliations:** 1 Department of Pharmacology, Faculty of Medicine, Yıldırım Beyazıt University, Ankara Turkey; 2 Department of Cardiology, Occupational and Environmental Diseases Hospital, Ankara Turkey; 3 Department of Family Medicine, Occupational and Environmental Diseases Hospital, Ankara Turkey; 4 Department of Biochemistry, Faculty of Medicine, Yıldırım Beyazıt University, Ankara Turkey; 5 Department of Pharmacology, Occupational and Environmental Diseases Hospital, Ankara Turkey; 6 Department of Toxicology, Council of Forensic Medicine, Branch Office for Ankara Turkey; 7 Department of Public Health, Faculty of Medicine, Yıldırım Beyazıt University, Ankara Turkey; 8 Department of Medical Biochemistry, Faculty of Medicine, Bozok University, Yozgat Turkey

**Keywords:** Lead exposure, endocan, diastolic function

## Abstract

**Background/aim:**

Lead can cause morphological and functional changes in heart, and inflammation and endothelial dysfunction in vasculature. Endocan, as a novel indicator of endothelial dysfunction, has been used for cardiovascular diseases. This study investigated the relationship between lead exposure, endocan levels, and diastolic functions.

**Materials and methods:**

A total of 51 lead-exposed workers without a known cardiovascular disease or risk factors and 54 healthy controls were enrolled. All participants underwent transthoracic echocardiography. Blood lead and serum endocan levels were analyzed.

**Results:**

Baseline demographic and clinical characteristics were found to be similar between groups. Median blood lead (32 vs 1.5 µg/dL, P < 0.001) and serum endocan levels (67 vs 57.1 pg/mL, P = 0.02) were significantly higher in the lead-exposed group. Serum endocan level showed a positive correlation with blood lead levels (r = 0.404, P = 0.003) in lead-exposed workers. Serum endocan level was an independent risk factor for increased E/E’ ratio (β = 0.704, P = 0.002) and left atrial volume index (β = 1.158, P = 0.011) and higher level of lead in blood was an independent risk factor for increased E wave (β = 8.004, P = 0.022) in lead-exposed workers.

**Conclusion:**

Worsened diastolic functions may be seen in the course of lead exposure. Due to sharing a similar mechanism, a higher serum level of endocan may be a valuable laboratory clue for impaired diastolic function in this population.

## 1. Introduction

Lead, a common environmental toxic agent, gives rise to plenty of cardiovascular diseases including ischemic heart disease, arterial hypertension, morphological, and functional changes in heart (1–3). Though its exposure is considered to be a risk factor for development of cardiovascular diseases primarily related to arterial hypertension, it is also capable of causing endothelial dysfunction (4).

Endocan, also known as endothelial cell-specific molecule-1, is a soluble proteoglycan expressed by vascular endothelial cells (5). Previous studies demonstrated that endocan has a key role in the pathological process of endothelial dysfunction, and serum endocan level increases in endothelium-dependent pathologies, inflammatory diseases, and malignancies (6). Recently, numerous studies have focused on the relationship between endocan and cardiovascular diseases (7–9).

The aim of this study was to investigate the relationship between blood lead levels, serum endocan levels, and diastolic functions measured by echocardiography in lead-exposed workers. 

## 2. Materials and methods

### 2.1. Study population

In the present cross-sectional study, 64 male workers occupationally exposed to lead who referred to our clinic were enrolled. Medical histories and occupational profiles of the patients were solicited. Physical examination, 12-lead surface electrocardiogram, laboratory and transthoracic echocardiography findings were also evaluated and recorded. Workers with ischemic heart disease, arterial hypertension defined as systolic blood pressure of >140 mmHg and diastolic blood pressure of >90 mmHg and/or taking anti-hypertensive medication, diabetes mellitus, cigarette smokers, and alcohol consumers were excluded. In order to eliminate the effects of any conduction disturbances or arrhythmias on diastolic functions, subjects suffered from them were also excluded. A total of 13 workers were excluded and of the remaining 51 workers included, 42 (82.3%) were employed in battery production and 9 (17.7%) were employed in metal recycling. Although multiple heavy metal exposure is generally seen concurrently, the study population was composed of lead exposure only. The control group included 54 healthy subjects with no previous history of cardiac disease. All subjects were males aged older than 18 years. This study complies with the Declaration of Helsinki, the locally appointed ethics committee has approved the research protocol, and informed consent has been obtained from the subjects.

### 2.2. Collection and analysis of biological samples

Blood samples were obtained at the end of work shift and were drawn in 10-mm red-capped tubes that did not contain gel (BD Vacutainer; Becton Dickinson Medical, Franklin Lakes, NJ, USA) for analysis of biochemical parameters. Lead levels were determined in whole blood samples using inductively coupled plasma-mass spectrometry (7700 series; Agilent Technologies, Inc., Santa Clara, CA, USA). Blood samples were digested using the microwave-induced acid method. Standard solution of lead was prepared by dilution of certified standard solutions (High Purity Standards, Charleston, SC, USA). Two-level quality control materials were used (Seronorm; Sera AS, Billingstad, Norway). Lead calibration curve ranged from 0 to 100 μg/dL. Limits of detection and quantification were 0.02 and 0.1 μg/dL, respectively. 

### 2.3. Determination of serum endocan levels 

Blood samples for endocan from subjects were also collected at the end of work shift and transferred to 16 × 100 mm tubes with red caps that did not contain gel (BD Vacutainer; Becton Dickinson Medical, Franklin Lakes, NJ, USA). Serum samples were separated after centrifugation at 1500 *g* for 10 min and stored at −80 °C until analysis. Serum endocan levels were measured with commercially available sandwich enzyme-linked immunosorbent assay (ELISA) kit with high sensitivity and specificity for detection of human endocan (Boster, Biological Technology Co. Ltd, Pleasanton, CA, USA).

### 2.4. Transthoracic echocardiography

Standard echocardiography imaging was performed according to the recommendations of the American Society of Echocardiography (10, 11). Left ventricular dimensions were obtained in parasternal long-axis view. Left ventricular ejection fraction was calculated according to the modified biplane Simpson’s rule. In the first instance, left atrial volume was measured using the biplane area-length method, it was then corrected for body surface area and redefined as left atrial volume index. 

Mitral inflow velocities were measured by pulsed wave Doppler from apical 4- chamber view positioned at the tips of mitral leaflets. Peak early filling velocity (E wave), peak filling velocity during atrial systole (A wave), E/A ratio, deceleration time of early filling velocity, and isovolumic relaxation time were calculated. Peak early diastolic tissue velocity (E’) of mitral annulus was measured from 4- chamber view. E/E’ ratio was calculated as E wave divided by E’.

### 2.5. Statistical analysis

Statistical analysis was performed using SPSS software (version 20.0; SPSS Inc., Chicago, IL, USA). Variables with normal distribution were analyzed using Kolmogorov–Smirnov test and presented as mean ± SD, while those without normal distribution were presented as median with minimum and maximum range. Categorical variables were presented as number and percentage. Continuous variables showing normal distribution were compared with t-test for independent variables, whereas for those showing no normal distribution, Mann–Whitney U test was used. Likewise, to test the possible correlation between variables with and without normal distribution, Pearson and Spearman correlation analysis were used, respectively. Stepwise linear regression analysis was used for multivariate regression analysis. Before this analysis, logarithmic transformation was applied on variables without normal distribution. 

## 3. Results

Baseline demographic, clinical and laboratory characteristics are shown in Table 1. No statistically significant difference was found between the groups regarding age, systolic and diastolic blood pressures, and laboratory parameters. Mean age of the lead-exposed group was 34.4 ± 7.2 years and that of the control groups was 33.0 ± 7.9 years. Median blood lead levels [32 µg/dL (range 13.3–78) vs 1.5 µg/dL (range 0.6–3.1), respectively, P < 0.001] and serum endocan levels [67 pg/mL (range 4.38–413.2) vs 57.1 pg/mL (0.79–354.9), respectively, P = 0.02] were found to be significantly higher in the group exposed to lead compared to the control group.

**Table 1:** Baseline demographic, clinical, laboratory, and serologic data of the lead-exposed and control groups.

Characteristics	Lead-exposed group (n = 51)	Control group(n = 54)	p
Age (years)	34.4 ± 7.2	33.0 ± 7.9	0.347
Systolic blood pressure (mmHg)	123.6 ± 14.2	121.9 ± 13.8	0.535
Diastolic blood pressure (mmHg)	76.5 ± 8.2	74.1 ± 7.8	0.127
Body mass index (kg/m2)	21.9 ± 2.1	22.0 ± 2.3	0.751
Hemoglobin (g/dL)	15.5 ± 1.24	15.7 ± 1.13	0.492
White blood cell count (/µL)	7343 ± 1649	7117 ± 1711	0.986
Neutrophil count (/µL)	4085 ± 1352	4089 ± 1179	0.551
Lymphocyte count (/µL)	2414 ± 750	2332 ± 661	0.246
Platelet count (/µL)	227880 ± 46144	229090 ± 44637	0.892
Fasting blood glucose (mg/dL)	91.8 ± 8.8	89.3 ± 9.4	0.166
Total cholesterol (mg/dL)	174.2 ± 35.8	186.3 ± 37.2	0.092
Low-density lipoprotein (mg/dL)	108.8 ± 30.8	116.1 ± 25.8	0.193
High-density lipoprotein (mg/dL)	45.2 ± 9.7	47.7 ± 9.3	0.169
Triglyceride (mg/dL)	142.0 ± 77.2	160.5 ± 60.3	0.177
Creatinine (mg/dL)	0.81 ± 0.11	0.85 ± 0.11	0.097
Alanine aminotransferase (U/L)	23.2 ± 11.0	23.4 ± 7.9	0.903
Aspartate aminotransferase (U/L)	20.1 ± 6.2	21.3 ± 7.6	0.375
Thyroid stimulating hormone (µIU/mL)	1.35 ± 0.79	1.54 ± 0.90	0.234
Triiodothyronine (pg/mL)	2.96 ± 0.50	2.81 ± 0.45	0.115
Thyroxine (ng/dL)	1.07 ± 0.21	1.16 ± 0.28	0.068
Erythrocyte sedimentation rate (mm/h)	6 [1–30]	9 [1–22]	0.148
Serum endocan level (pg/mL)	67 [4.38–413.2]	57.1 [0.79–354.9]	0.020
Blood lead level (µg/dL)	32 [13.3–78]	1.5 [0.6–3.1]	<0.001

Transthoracic echocardiographic measurements of the groups are shown in Table 2. Left ventricular ejection fractions, left ventricular end-diastolic diameters, and left ventricular end-systolic diameters were similar and normal in the two groups. In terms of diastolic functions parameters, no statistically significant difference was found between the groups regarding A wave, isovolumic relaxation time, and deceleration time. However, E wave (104.1 ± 9.5 cm/s vs 108.2 ± 10.3 cm/s, P = 0.03), E/A ratio (1.06 ± 0.16 vs 1.15 ± 0.23, P = 0.011) and E’ wave (10.9 ± 1.9 cm/s vs 12.3 ± 1.6 cm/s, P = 0.001) were found to be lower in the lead-exposed group than that of controls. On the other hand, E/E’ ratio (9.8 ± 1.8 vs 8.9 ± 1.2, P = 0.006) and left atrial volume index (32.3 ± 3.5 mL/m2 vs 30.9 ± 3.0 mL/m2, P = 0.035) were found to be higher in the lead-exposed group than that in the control group.

**Table 2:** Transthoracic echocardiographic parameters of the groups.

Characteristics	Lead-exposed group (n = 51)	Control group (n = 54)	p
Left ventricular end diastolic diameter (mm)	46.4 ± 2.3	46.1 ± 3.0	0.643
Left ventricular end systolic diameter (mm)	28.6 ± 2.8	28.5 ± 3.0	0.823
Left ventricular ejection fraction (%)	64.8 ± 3.0	64.4 ± 2.8	0.426
E wave (cm/s)	104.1 ± 9.5	108.2 ± 10.3	0.030
A wave (cm/s)	100.1 ± 15.2	95.8 ± 14.1	0.138
E/A ratio	1.06 ± 0.16	1.15 ± 0.23	0.011
E’ wave (cm/s)	10.9 ± 1.9	12.3 ± 1.6	0.001
E/E’ ratio	9.8 ± 1.8	8.9 ± 1.2	0.006
Isovolumic relaxation time (ms)	82.3 ± 7.7	82.0 ± 7.3	0.970
Deceleration time (ms)	183.2 ± 15.2	182.0 ± 14.5	0.686
Left atrial volume index (mL/m2)	32.3 ± 3.5	30.9 ± 3.0	0.035

**Figure 1:**
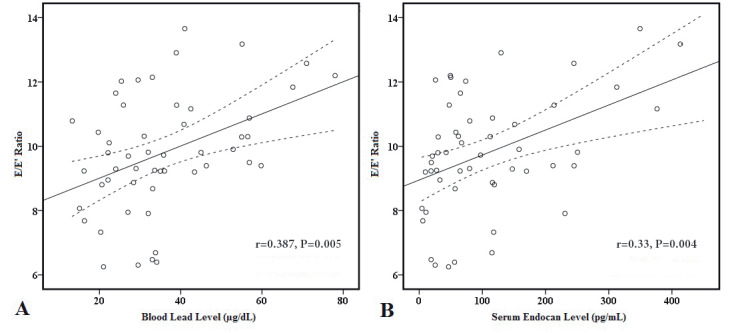
Graphics of correlation between E/E’ ratio and (A) blood lead level and (B) serum endocan level. Solid lines represent the correlation slope, dotted lines represent 95% confidence intervals.

**Figure 2:**
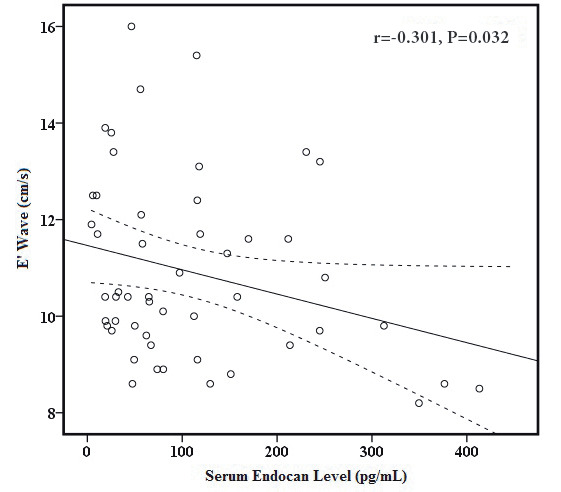
Graphic of correlation between E’ wave and serum endocan level. Solid line represents the correlation slope, dotted lines represent 95% confidence intervals.

**Figure 3:**
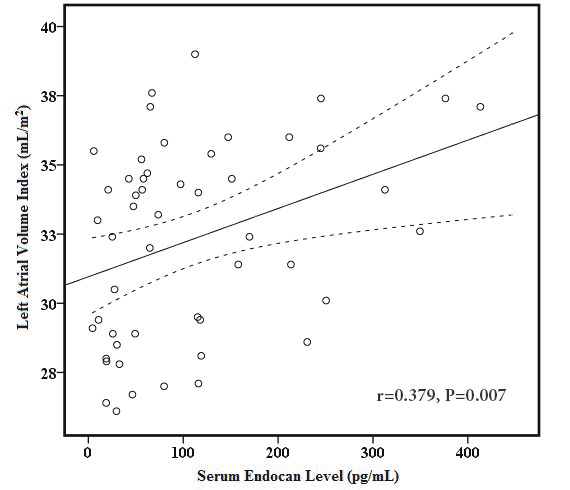
Graphic of correlation between left atrial volume index and serum endocan level. Solid line represents the correlation slope, dotted lines represent 95% confidence intervals.

**Figure 4:**
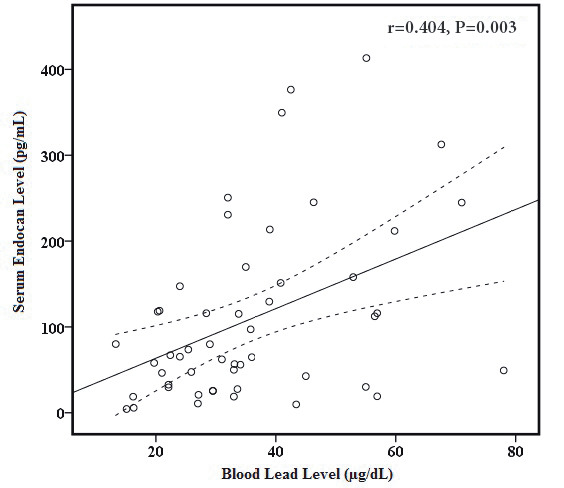
Graphic of correlation between serum endocan level and blood lead level. Solid line represents the correlation slope, dotted lines represent 95% confidence intervals.

In correlation analysis in the lead-exposed group (Table 3), E wave (r = 0.339, P = 0.015 and r = 0.382, P = 0.006, respectively) and E/E’ ratio (r = 0.387, P = 0.005, Figure 1A and r = 0.393, P = 0.004, Figure 1B, respectively) were to be significantly correlated with both blood lead levels and serum endocan level. Serum endocan level was negatively correlated with E’ wave (r = −0.301, P = 0.032, Figure 2) and positively correlated with left atrial volume index (r = 0.379, P = 0.007, Figure 3). Additionally, serum endocan level showed a positive correlation with blood lead levels (r = 0.404, P = 0.003, Figure 4) While A wave, E/A ratio, isovolumic relaxation time, and deceleration time were not found to be correlated with blood lead and serum endocan levels. 

**Table 3:** Correlation analysis results between blood lead level, endocan level, and echocardiographic parameters of diastolic functions in the lead-exposed workers.

Characteristics	Blood lead level(µg/dL)		Serum endocan level(pg/mL)	
	r	P	r	P
E wave (cm/s)	0.339	0.015	0.382	0.006
A wave (cm/s)	0.258	0.061	0.097	0.497
E/A ratio	−0.087	0.545	0.092	0.519
E’ wave (cm/s)	−0.251	0.076	−0.301	0.032
E/E’ ratio	0.387	0.005	0.393	0.004
Isovolumic relaxation time (ms)	−0.024	0.866	−0.033	0.819
Deceleration time (ms)	−0.182	0.201	0.014	0.925
Left atrial volume index (mL/m2)	0.0190	0.181	0.379	0.007

In multivariate stepwise linear regression analysis in the lead-exposed group (Table 4), serum endocan level was found to be an independent risk factor for increased E/E’ ratio (β = 0.704, P = 0.002) and left atrial volume index (β = 1.158, P = 0.011). Additionally, a higher level of lead in blood was found to be an independent risk factor for increased E wave (β = 8.004, P = 0.022). 

**Table 4:** Multivariate stepwise linear regression analysis results for determination of predictors of E/E’ ratio, E wave, and left atrial volume index in the lead-exposed workers.

	β ± SE	95% Confidence interval		P
		Lower	Upper		
E/E’ Ratio				
Serum endocan level (pg/mL)*	0.704 ± 0.221	0.260	1.148	0.002
R^2^ = 0.415, Adjusted R^2^ = 0.155, P = 0.00				
E wave				
Blood lead level (µg/dL)*	8.004 ± 3.055	1.865	14.143	0.012
R^2^ = 0.351, Adjusted R^2^ = 0.105, P = 0.012				
Left atrial volume index				
Serum endocan level (pg/mL)*	1.158 ± 0.439	0.277	2.039	0.011
R^2^ = 0.353, Adjusted R^2^ = 0.107, P = 0.011				

β: Regression coefficients; SE: Standard error; R: Model fit. * Logaritmic transformation was applied to blood lead level and serum endocan level in order to obtain normal distribution.

## 4. Discussion

In the present study, diastolic functions on echocardiography were evaluated. In the lead-exposed workers, compared to control subjects, while E/E’ ratio and left atrial volume index were found to be higher, E wave, E/A ratio, and E’ wave were found to be lower. Moreover, serum endocan levels as a novel biomarker for vascular inflammation and endothelial dysfunction were increased in these workers. Additionally, significant correlations were found between diastolic parameters, serum endocan and blood lead levels.

The leading negative effects of lead on the cardiovascular system show themselves in blood pressure profile (2) and the atherosclerotic process in arterial system (12). However, occupational exposure to lead may also contribute to impairment of left ventricular diastolic dysfunction but there are only a few studies in which echocardiographic examination was performed. Poreba et al. (3) demonstrated worsened systolic and diastolic functions on echocardiography in patients with occupational exposure to lead. Deceleration time and E/E’ ratio in these patients were observed to be higher compared to the control subjects, whereas the E/A ratio and E’ wave were lower. Similarly, in the present study, E/E’ ratio was also found to be higher and E’ wave and E/A ratio were also found to be lower in lead-exposed workers. In contrast, left atrial volume index was also found to be higher in lead-exposed workers in the present study and deceleration time and systolic functions did not show a significant difference between the lead-exposed workers and the control subjects. Additionally, high blood zinc protoporphyrin concentration, which is an indicator of lead exposure, was demonstrated to be a negatively independent risk factor for E’ wave in the above-mentioned study. In the present study, blood lead levels were found to be independent risk factors for E wave. 

It has been suggested that mechanisms involved in ventricular diastolic dysfunction and endothelial dysfunction might be similar (13). Increased oxidative stress, decreased nitric oxide synthesis, imbalance between vasomotor/vasodilator substances and excessive sympathetic activity have been implicated the same mechanisms involved in development of both endothelial dysfunction and left ventricular diastolic dysfunction (14–16). Ma et al. suggested that endothelial dysfunction was related to left ventricular diastolic dysfunction in patients with coronary heart disease (13). Elesber et al. showed that coronary endothelial dysfunction was independently associated with impaired relaxation in patients with normal ejection fraction in the absence of occlusive coronary artery disease and heart failure (17). Additionally, beneficial effects of angiotensin-converting enzyme inhibitors and beta blockers on diastolic dysfunction and endothelial dysfunction also suggest that there may be a similar mechanism in the development of myocardial injury and endothelial impairment (18,19). In the case of lead exposure, tissue damage might be seen through similar mechanisms (20). Lead exposure promotes oxidative stress and inflammation, increases endothelin production and vasoconstrictor prostaglandins, and reduces nitric oxide availability and vasodilator prostaglandins (4). Thus, endothelial dysfunction may play a central role in the relationship between lead exposure and diastolic dysfunction (13). 

Endocan was increased by many inflammatory diseases and used as a biomarker for ongoing vascular inflammation and endothelial dysfunction. Increased serum endocan levels were observed in various clinical settings characterized by endothelial dysfunction such as hypertension (21), obstructive coronary artery disease (22), acute coronary syndrome (9,23), and diabetes mellitus (24, 25). In the present study, serum endocan level was analyzed in the case of lead exposure, and endothelial dysfunction as main link between lead exposure and diastolic dysfunction has been evaluated by serum endocan level for the first time. Serum endocan levels were demonstrated to be higher and independent risk factors for increased E/E’ ratio and left atrial volume index in the lead-exposed group. Furthermore, serum endocan and blood lead levels showed strong correlation in the lead-exposed workers. It may be due to having similar effects on the same milieu. Previous studies have shown that lead reduces endothelial cell growth, impedes endothelial repair, and promotes proinflammatory cytokines (4,26). On the other hand, endocan has been implicated in leukocyte–endothelium interaction and reported as a marker for endothelial dysfunction (27). 

In contrast with previous studies, the subjects of the present study were normotensive and none of them had any etiology which might result in diastolic dysfunction since individuals with hypertension and structural heart diseases were excluded. Results of the present cross-sectional study suggest that lead exposure can be assumed as a risk factor for changes in diastolic functions regardless of blood pressure status and the presence of a triangular relationship between lead exposure, endothelial dysfunction evaluated by serum endocan level and diastolic dysfunction. 

## 4.1. Limitations of the study

Lead accumulates almost entirely in bone. In the present study, only blood lead levels were analyzed. The workers were evaluated with regard to their “lead exposure” independent of their blood lead levels, so there was no cut-off value for blood lead concentration. Study groups were extremely homogeneous due to tight exclusion criteria that might cause to reduce representing real-world conditions. In addition, workplace testing was not analyzed. Finally, this study is cross-sectional in design and subjects were not followed up in terms of cardiovascular end-points. These limitations make it so hard to speculate long-term cardiovascular complications. The findings of this study will need confirmation with larger studies.

Impairment in diastolic parameters on echocardiography may be seen in lead-exposed individuals and serum endocan level may be a useful marker for monitoring the development of cardiovascular complications in the case of lead exposure even in the early course of involvement. Other endothelial-dependent cardiovascular complications in lead exposure are needed to be studied.
